# Development and validation of an eight-step flowchart based on the CAM-ICU: a quick and highly adaptable tool to determine the presence of delirium in ICU patients

**DOI:** 10.1186/cc9755

**Published:** 2011-03-11

**Authors:** IJ Zaal, LM Peelen, D Van Dijk, AJ Slooter

**Affiliations:** 1 University Medical Centre Utrecht, the Netherlands

## Introduction

Delirium is a frequent and serious disorder in the ICU. Several tools have been developed for standardized delirium testing, of which the Confusion Assessment Method for the ICU (CAM-ICU) is the best validated and most widely used. The main limitations of the CAM-ICU are, however, that it is a very brief assessment of a highly fluctuating disorder, and that the test may lack sensitivity when administered in daily practice. For research purposes, we extended the CAM-ICU.

## Methods

This ongoing prospective validation study was performed in a 12-bed, mixed adult ICU of the University Medical Centre Utrecht. All patients admitted to the ICU were assessed daily and independently on delirium by two means: a junior doctor or neurologist (gold standard); and an eight-item flowchart, based on the CAM-ICU, the reports of the bedside nurses and the administration of haloperidol. Exclusion occurred when patients remained unresponsive (RASS <-3) during admission or when they were unable to understand Dutch and English. With both assessment methods, patients were classified as either awake without delirium, delirious for one or more moments in the past 24 hours, or comatose during the whole past 24 hours.

## Results

A total of 55 patients (35 men, 63.6%; mean age 60.0, SD 17.9; mean APACHE II score 18.7, SD 6.1) were included and 379 assessments were made. The form, which excludes patients with neurological pathology for further analysis, showed a sensitivity of 85%, a specificity of 88%, a positive predictive value of 81% and a negative predictive value of 91%.

## Conclusions

While the CAM-ICU is a tool to assess delirium during a brief observation period, this extension can be used to classify the presence of delirium in the previous hours in an ICU where the CAM-ICU is already implemented. The tool appeared to be easy in use and highly adaptable with good test characteristics.

